# Neurophysiological assessment of juvenile parkinsonism due to primary monoamine neurotransmitter disorders

**DOI:** 10.1007/s00702-022-02527-z

**Published:** 2022-07-12

**Authors:** Massimiliano Passaretti, Luca Pollini, Giulia Paparella, Alessandro De Biase, Donato Colella, Luca Angelini, Serena Galosi, Filippo Manti, Andrea Guerra, Vincenzo Leuzzi, Alfredo Berardelli, Matteo Bologna

**Affiliations:** 1 Department of Human Neuroscience, Sapienza University of Rome, Viale dell’Università 30, 00185 Rome, Italy; 2 Child Neurology and Psychiatry, Department of Human Neuroscience, Sapienza University of Rome, Rome, Italy; 3 IRCCS Neuromed, Pozzilli, IS Italy

**Keywords:** Inherited monoamine neurotransmitter disorders, Juvenile parkinsonism, Transcranial magnetic stimulation, Bradykinesia, Finger tapping

## Abstract

No studies have investigated voluntary movement abnormalities and their neurophysiological correlates in patients with parkinsonism due to inherited primary monoamine neurotransmitter (NT) disorders. Nine NT disorders patients and 16 healthy controls (HCs) were enrolled. Objective measurements of repetitive finger tapping were obtained using a motion analysis system. Primary motor cortex (M1) excitability was assessed by recording the input/output (I/O) curve of motor-evoked potentials (MEP) and using a conditioning test paradigm for short-interval intracortical inhibition (SICI) assessment. M1 plasticity-like mechanisms were indexed according to MEPs amplitude changes after the paired associative stimulation protocol. Patient values were considered abnormal if they were greater or lower than two standard deviations from the average HCs value. Patients with aromatic amino acid decarboxylase, tyrosine hydroxylase, and 6-pyruvoyl-tetrahydropterin synthase defects showed markedly reduced velocity (5/5 patients), reduced movement amplitude, and irregular rhythm (4/5 patients). Conversely, only 1 out of 3 patients with autosomal-dominant GTPCH deficiency showed abnormal movement parameters. Interestingly, none of the patients had a progressive reduction in movement amplitude or velocity during the tapping sequence (no sequence effect). Reduced SICI was the most prominent neurophysiological abnormality in patients (5/9 patients). Finally, the I/O curve slope correlated with movement velocity and rhythm in patients. We provided an objective assessment of finger tapping abnormalities in monoamine NT disorders. We also demonstrated M1 excitability changes possibly related to alterations in motor execution. Our results may contribute to a better understanding of the pathophysiology of juvenile parkinsonism due to dopamine deficiency.

**Supplementary Information:**

The online version contains supplementary material available at 10.1007/s00702-022-02527-z.

## Introduction

Inherited primary monoamine neurotransmitter (NT) disorders are a group of inborn errors of metabolism, resulting in dopamine, serotonin, norepinephrine, and epinephrine metabolism deficiency (Morales-Briceño et al. 2020; Leuzzi et al. 2021). These conditions include disorders of biogenic amine synthesis, such as tyrosine hydroxylase (TH) and aromatic amino acid decarboxylase (AADC) defects, and disorders of tetrahydrobiopterin cofactor metabolism, comprising autosomal-dominant (AD) GTPCH deficiency and 6-pyruvoyl-tetrahydropterin synthase (PTPS) deficiency (Morales-Briceño et al. 2020; Leuzzi et al. 2021).

From a clinical point of view, parkinsonism and dystonia-parkinsonism with diurnal fluctuation and oculogyric crises can be present to a variable extent in adult patients with NT disorders, with symptom onset during infancy with generalized rigid- or hypotonic hypokinesia, developmental delay and oculogyric crises, or later, during childhood, with a pure dystonia with progressive generalization. All these conditions, except for AADC defect, respond to the treatment with dopamine precursors or mimics (Ng et al. 2015; Galosi et al. 2020; Leuzzi et al. 2021). From a pathophysiological standpoint, motor symptoms in patients with monoamine NT disorders are thought to primarily result from reduced dopamine synthesis and altered basal ganglia activity (Morales-Briceño et al. 2020; Leuzzi et al. 2021). A few neurophysiological investigations have explored the activity of cortical motor areas using transcranial magnetic stimulation (TMS) techniques, and have shown variable results regarding primary motor cortex (M1) excitability in patients with dopa-responsive dystonia (Huang et al. 2006; Hanajima et al. 2007; Weissbach et al. 2015, 2021, 2022). However, no TMS studies have been performed in patients with other types of monoamine NT disorders, and no studies have correlated possible movement execution abnormalities with changes in M1 excitability and plasticity. A better understanding of movement execution and cortical activity in patients with monoamine NT disorders would help to better understand the pathophysiology of parkinsonism in these conditions.

In the present study, we objectively investigated possible alterations of finger tapping movements, which is now the most widely used task for bradykinesia assessment in clinical practice, in patients with primary monoamine NT disorders, including AADC, TH, PTPS defects, and AD-GTPCH defects. We also assessed cortical excitability and plasticity measures of M1 using TMS techniques. Finally, we investigated possible correlations between movement abnormalities and TMS findings in patients.

## Materials and methods

### Participants

Nine patients with primary monoamine NT disorders (5 females, mean age ± 1 standard deviation-SD: 29.6 ± 11.37; Table 1) and 16 HCs (6 females, mean age ± 1 SD: 27 ± 3.74) were enrolled in the study. All participants were older than 18 years and all were right-handed, as evaluated by the Edinburgh Handedness Inventory. Patients were consecutively recruited from the Child Neurology and Psychiatry Unit, Department of Human Neuroscience, Sapienza University of Rome, Italy. They all had a diagnosis of primary monoamine NT disorders, genetically confirmed and/or with proven cerebrospinal fluid (CSF) monoamine level abnormalities (Ng et al. 2015) (Table 1). Three patients had a diagnosis of AADC (DYT-DDC) (patients 1, 2, and 3), one had a diagnosis of TH defect (DYT5b, DYT/PARK-TH) (patient 4), one had a diagnosis of PTPS defect (DYT/PARK-PTS) (patient 5), and three patients had a diagnosis of AD-GTPCH due to *GCH1* gene mutations (DYT5a, DYT/PARK-GCH1) (patients 6, 7, and 8). Finally, one patient (patient 9) presented with an early onset parkinsonism associated with a low level of homovanillic acid (HVA) in CSF (Table 1). This patient carries two variants of unknown significance in the *TH* gene [c.772G > A (p.Glu258Lys); c.360 G > A (p.Arg120 =)] and lacks of a definitive genetic diagnosis. All patients underwent a complete neurological examination, including the administration of the motor section (part III) of the Movement Disorder Society-sponsored revision of the Unified Parkinson’s Disease Rating Scale (MDS-UPDRS) (Goetz et al. 2008). The cognitive level was assessed through a formal evaluation conducted with the Wechsler Adult Intelligence Scale-Revised IV (WAIS-IV). Due to verbal impairment, Leiter International Performance Scale (Leiter III) was administered to patients 1 and 3. All patients were on pharmacological treatment since the diagnosis (Table 1). All participants provided written informed consent to participate in the study. Experimental procedures were approved by the local ethics committee and performed according to the Declaration of Helsinki.

**Table 1:** Demographic and clinical data of patients with monoamine neurotransmitter (NT) disorders

ID Sex	Age (years)	Age at onset (months)	Disease (Gene)	HVA in CSF at diagnosis*	5-HIAA in CSF at diagnosis*	Genotype	MDS-UPDRS-III	IQ	Therapy	LEDD
1F	25	4	AADCD (*DDC*)	**57** (211–371)	**14** (105–299)	p.[Ser250Phe];[Ser250Phe]	115	33	l-Dopa/carbidopa Rotigotine; selegiline	340
2F	41	12	AADCD (*DDC*)	169 (98–450)	50 (45–135)	p.[Tyr37Thrfs*5];[Phe237Ser]	14	61	Pramipexole; selegiline	178
3 M	19	12	AADCD (*DDC*)	**85.1** (144–801)	**21.6** (86–78)	p.[Cys281Trp]; [Met362Thr]	36	40	Rotigotine; selegiline	280
4 M	32	4	THD (*TH*)	**68** (148–434)	143 (45–135)	p.[Leu510Gln];[Gly414Arg]	31	63	l-Dopa/carbidopa; Selegiline	120
5 M	44	12	PTPS (*PTS*)	**30** (98–450)	69 (45–135)	p.[Arg9Cys];[Pro87Leu]	24	51	Rasagiline; Sapropterin dihydrochloride	100
6F	18	36	GTPCHD (*GCH1*)	188 (137–582)	74 (68–220)	Exon 1 Del	8	120	l-Dopa/carbidopa	225
7F	46	216	GTPCHD (*GCH1*)	NA	NA	Exon 1 Del	2	129	–	–
8F	23	9	GTPCHD (*GCH1*)	229 (148–434)	143 (45–135)	p.[M211fs]	4	63	l-Dopa/carbidopa	200
9 M	19	156	TH (TH)	**92.29** (148–434)	132.98 (68–115)	p.[Glu258Lys; Arg120 =]**	45	51	l-Dopa/carbidopa l-Dopa/carbidopa	250
*Mean* *SD*	29.60 11.37	51.20 78.53					31 34.84	67.88 31.80		

Abnormal HVA and 5-HIAA values in CSF are in bold

*CSF* cerebrospinal fluid, *HVA* Homovanillic acid, *5-HIAA* 5-Hydroxyindoleacetic acid, *MDS-UPDRS-III* movement disorder society-sponsored revision of the unified Parkinson’s disease rating scale, part III, *IQ* intellectual quotient, *LEDD* l-dopa equivalent daily dose, *M* male, *F* female, *SD* standard deviation, *NA* not available

*Diagnostic value expressed in nmol/L

**Identified variants are in cys and of uncertain significance. The case is considered undiagnosed

### Kinematic assessment

Three 15 s repetitive finger tapping trials were recorded from the more affected side in patients and from the dominant hand in HCs while participants were comfortably seated in a chair. Before kinematic recordings, one practice trial was allowed for the participants to become familiar with the task. A period of 45–60 s of rest was given between acquisition trials to avoid fatigue. Kinematic recordings were performed using an optoelectronic system (SMART motion system, BTS Engineering, Italy) composed of three infrared cameras (sampling rate of 120 Hz). These cameras recorded the movements of reflective markers with a 5 mm diameter and of negligible weight taped to participants’ hands. Two markers were placed on the tips of the index finger and thumb. Three other markers were placed on the hand to define a reference plane that was used to exclude possible contamination due to unwanted hand movements from repetitive finger tapping (Bologna et al. 2016).

Movement analysis was performed using a dedicated software system (SMART Analyzer, BTS Engineering, Italy). To quantify repetitive finger movement kinematics, we used linear regression techniques to calculate the intercept, which reflects the movement amplitude (degree) and velocity (degree/s), and the slope, which reflects the amplitude and velocity decrement during movement repetition. Movement rhythm was also measured by the coefficient of variation (CV) of the inter-tap intervals. Higher CV values represented a lower regularity of repetitive movements (Bologna et al. 2016). The average of the three recording trials was considered for the analysis.

### TMS techniques and electromyographic recordings

Single- and paired-pulse TMS was delivered using two Magstim magnetic stimulators (Magstim Company, UK) connected to a figure-of-eight-shaped coil, with the intersection of the coil held tangentially to the scalp and the coil handle positioned at a ~ 45° angle from the midline pointing backward. We first defined the hotspot of the abductor pollicis brevis (APB) muscle, i.e., the optimal scalp position to elicit motor-evoked potentials (MEP) of maximal amplitude in the muscle. We then determined the resting and active motor thresholds (RMT and AMT) to the nearest 1% of the maximal stimulator output (Rossini et al. 2015). To probe M1 excitability, we measured the MEP input–output (I/O) curve. We delivered 40 single-pulse stimuli in groups of 10 at four stimulation intensities, ranging in 20% increments from 100 to 160% of RMT. The intensity order was chosen randomly to avoid possible hysteresis effects (Möller et al. 2009). We also assessed short-interval intracortical inhibition (SICI) using paired-pulse TMS with a subthreshold conditioning stimulus (90% AMT) and a supra-threshold test stimulus (1 mV MEP). We used interstimulus intervals (ISIs) of 2 and 4 ms between the conditioned stimulus (CS) and test stimulus (TS) (Rossini et al. 2015). Ten trials were acquired for each ISI. To study cortical plasticity, paired associative stimulation (PAS) was delivered over M1 contralateral to the more affected side in patients and to the dominant side in HCs (Kojovic et al. 2015). PAS was composed of 200 electrical stimuli, delivered to the median nerve at the wrist by means of a Digitimer DS7 (Digitimer, UK), paired with TMS stimuli (adjusted to 1 mV MEP intensity), delivered over the contralateral APB hotspot (rate 0.25 Hz, electrical stimulation intensity 2–3 times the perceptual threshold) (Kojovic et al. 2015). The electrical conditioning stimulus preceded each TMS stimulus at an ISI of 21.5 ms (Kojovic et al. 2015).

Electromyography (EMG) activity was recorded from the APB muscle of the more affected side in patients and of the dominant side in HCs, using surface electrodes taped in a belly-tendon montage. EMG signals were amplified and filtered (20 Hz-1 kHz) using Digitimer D360 (Digitimer, UK). EMG signals were recorded and stored on a laboratory PC (sampling rate of 5 kHz) through an analog–digital converter AD1401 plus (Cambridge Electronic Design, UK) for subsequent offline analyses, which were performed using dedicated software (Signal^®^ version 4.00, Cambridge Electronic Design, UK). Peak-to-peak MEP amplitude was measured within a time window of 20–40 ms after the TMS artifact. Traces with background EMG activity exceeding 100 µV in the 200 ms time window preceding the TMS artifact were rejected online. The steepness of the I/O MEP curve (i.e., the slope of the regression line across the scatterplot of the MEP amplitude (*Y* axis) vs. the stimulation intensity (*X* axis)) was calculated. SICI was expressed as the ratio between the amplitude of conditioned and unconditioned MEP. PAS effect was measured as the normalized percent ratio of post-PAS single-pulse MEP amplitude to pre-PAS values.

### Experimental design

Participants underwent one experimental session. Patients were studied after overnight withdrawal (at least 12 h) of their medication. Kinematic recordings of finger tapping and TMS measures of corticospinal and intracortical excitability were collected at baseline (T0). To assess M1 plasticity, we then performed the PAS protocol and recorded M1 excitability changes at three time points: T1 (5 min after PAS), T2 (15 min after PAS), and T3 (30 min after PAS) using single-pulse TMS. Twenty MEP were recorded at 1-mV intensity at each measurement time point (including T0).

### Statistical analysis

Age and gender differences between patients and HCs were evaluated using Mann–Whitney *U* and Fisher’s exact tests, respectively. Kinematic and TMS features of patients, including motor thresholds, I/O slope, SICI average (mean between 2 and 4 ms ISIs), and PAS average (mean MEP amplitude recorded at T1, T2, and T3), were compared with those of HCs. Patient values were considered abnormal if they were more or less than 2 SDs from the average HCs value. To test possible relationships between TMS and kinematic data, we used Spearman’s rank correlation. Notably, correlations were assessed using the data that were most frequently altered in patients. All results are shown as mean values ± SD unless otherwise indicated. Data were analyzed using STATISTICA^®^ (TIBCO Software Inc., Palo Alto, California, USA).

## Results

All study participants completed finger tapping kinematic recordings. One patient (patient 4) did not provide consent to undergo the TMS procedures. None of the participants reported adverse effects during the experiments. No difference was found in terms of age or gender distribution between patients and HCs (*p* values = 0.76 and 0.23, respectively). Clinical assessment showed that bradykinesia was evident in patient 1, 2, 3, 4, 5 and 9, while it was very mild in patient 6, and questionable in patient 7 and 8. Rigidity was detected in three patients (patients 1, 4, and 9), while rest tremor was only observed in one patient (patient 9). The MDS-UPDRS part III mean score ± SD in patients was 31 ± 34.84. The mean ± SD WAIS-IV score in patients was 67.88 ± 31.8.

Pretreatment biochemical profile (Table 1) showed abnormally low levels of HVA in CSF of 4 out of 5 patients with recessive disorders and normal level in both the patients with AD-GTPCH who underwent CSF examination.

### Finger tapping kinematics

Kinematic values from patients are shown in (Table 2). The prominent motor abnormality in patients was reduced movement velocity, which was observed in 7 out of 9 patients (77.7%). Reduced movement amplitude was also found in 6 out of 9 patients (66.6%). Similarly, 6 patients (66.6%) had higher CV values as compared to HCs, meaning that finger tapping was characterized by rhythm irregularity. The number of movements was reduced in 4 out of 9 patients (44.4%). Finally, velocity and amplitude slopes during movement repetition were within the normal range, indicating no sequence effect in patients. Notably, most patients with slow movement velocity (5/7, 71.4%) showed both movement amplitude and rhythm alterations. One patient (patient 6) had low velocity and amplitude values with normal CV values. Conversely, one patient (patient 3) had slow movement velocity, altered CV, and normal movement amplitude.

**Table 2:** Kinematic variables of finger tapping in patients with monoamine neurotransmitter (NT) disorders

	N. mov (49.24–77.80)	CV (0.021–0.144)	Amplitude (37.74–70.05)	Velocity (944.48–1572.76)	Amplitude slope (− 0.40–0.21)	Velocity slope (− 10.72 to − 3.52)
Pt 1	7	**0.224**	**31.41**	**491.1**	**0.708**	− 3.26
Pt 2	49.33	0.118	**28.46**	**598.52**	− 0.029	− 3.90
Pt 3	**32.667**	**0.37**	41.52	**562.51**	− 0.07	− 0.60
Pt 4	72.67	0.252	**12.89**	**396.87**	− 0.022	− 6.31
Pt 5	**45.33**	**0.175**	**25.68**	**734.06**	− 0.031	− 4.32
Pt 6	49.67	0.055	**27.65**	**821.38**	0.074	− 2.95
Pt 7	55	0.1	58.95	1020.56	0.030	− 9.65
Pt 8	**21**	**0.159**	78.1	986.9	− 0.081	− 4.61
Pt 9	**35**	**0.245**	**21.57**	**239.49**	**− 0.286**	− 6.31

For each parameter, the upper and lower limits of 2 standard deviations (SDs) from the mean control value are indicated in branches. Patient values greater or lower than 2 SDs from the average control value were considered abnormal and are in bold. *N. mov* number of movements; *CV* coefficient of variation; Amplitude is expressed in degrees. Velocity is expressed in degrees/s. Amplitude slope is expressed in degrees/n. mov. Velocity slope is expressed in (degrees/s)/n. mov

**Fig. 1:**
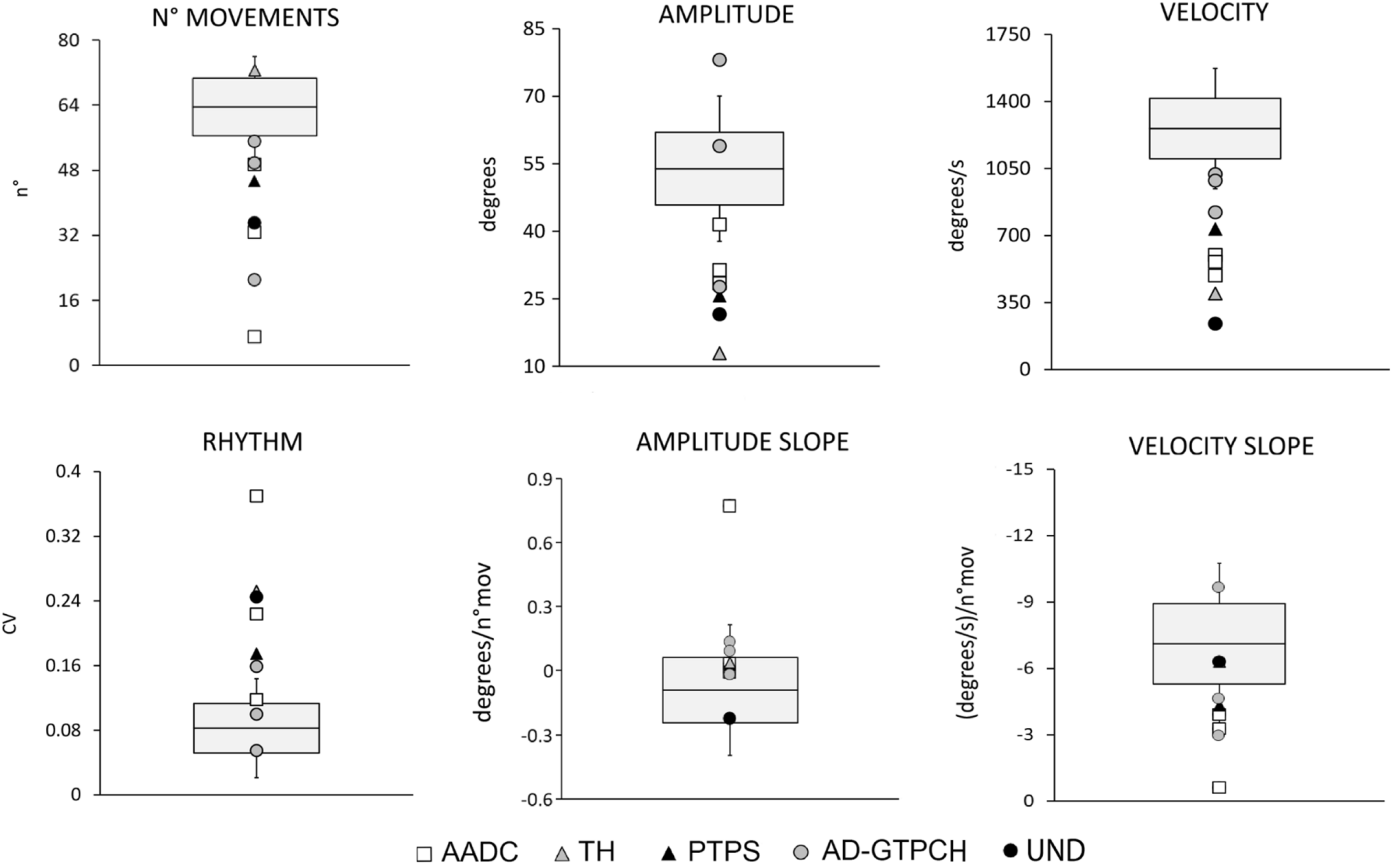
Kinematic variables of repetitive finger movements in patients with monoamine neurotransmitter (NT) disorders and healthy controls (HCs). *N. mov* number of movements, *CV* coefficient of variation. Horizontal lines denote average values in HCs. Boxes contain the mean value ± 1 standard deviation (SD) of the mean in HCs. Whiskers contain the mean value ± 2 SDs of the mean in HCs. White squares indicate individual data from patients with aromatic amino acid decarboxylase (AADC) deficiency (Patients 1–3). The grey triangle indicates data from the patient with tyrosine hydroxylase (TH) defect (Patient 4). The black triangle indicates data from the patient with 6-pyruvoyl-tetrahydropterin synthase (PTPS) defect (Patient 5). Grey circles indicate individual data from patients with autosomal-dominant GTPCH deficiency (AD-GTPCH) (Patients 6–8). The black circle indicates data from the patient with early onset parkinsonism associated with a low level of homovanillic acid (HVA) in cerebrospinal fluid but no definitive genetic diagnosis (Patient 9). Note that all AADC patients and those affected by TH and PTPS defects showed markedly abnormal kinematic parameters, including low movement velocity (5/5 patients), low movement amplitude (4/5, patients 1, 2, 4, 5), and high CV values (4/5, patients 1, 3, 4, 5). Namely, they performed slow, hypokinetic, and irregular movements. None of the patients showed a progressive reduction in movement amplitude or velocity during the tapping sequence (no sequence effect). Conversely, most patients with AD-GTPCH deficiency (2/3, 66.6%) showed normal kinematic parameters, and only 1 had slightly lower values of movement amplitude and velocity as compared to HCs, though he showed normal CV values. Finally, patient 9 showed markedly altered movement parameters

When considering patient diagnoses (Fig. 1), we found that all AADC patients (patients 1, 2, and 3) and those affected by TH and PTPS defects (patients 4 and 5) showed markedly abnormal kinematic parameters, including low movement velocity (5/5 patients), low movement amplitude (4/5, patients 1, 2, 4, 5), and high CV values (4/5, patients 1, 3, 4, 5). Namely, they performed slow, low amplitude, and irregular movements. Conversely, most patients with AD-GTPCH deficiency (2/3, 66.6%) showed normal kinematic parameters, and only 1 (patient 6) had slightly lower amplitude and velocity values as compared to HCs, though he showed normal movement rhythm. Finally, patient 9, whose genetic diagnosis was not available, showed markedly altered movement parameters (Fig. 1).

### TMS measurements

RMT, AMT, and single MEP amplitude in patients were within the normal range (Table 3).

**Table 3:** Transcranial magnetic stimulation (TMS) variables in patients with monoamine neurotransmitter (NT) disorders

	RMT (30–62.3)	AMT (18.8–48.8)	1 mV MEP (0.48–1.38)	I/O slope (0.2–0.8)	SICI (0.24–0.75)	PAS (103.43–152.12)
Pt 1	48	40	0.86	0.77	**1.02**	114.69
Pt 2	40	32	0.64	0.29	**0.91**	**96.58**
Pt 3	45	41	1.31	**1.67**	0.26	**194.81**
Pt 4	–	–	–	–	–	–
Pt 5	53	40	0.81	0.48	**1.05**	**161.9**
Pt 6	44	40	0.50	**0.03**	0.49	122.22
Pt 7	55	48	0.77	**0.19**	**0.86**	**98.71**
Pt 8	50	38	0.77	**0.17**	**0.92**	**93.4**
Pt 9	55	46	1.19	0.31	0.43	113.9

For each parameter, the upper and lower limits of 2 standard deviations (SDs) from the mean control value are indicated in branches. Patient values greater or lower than 2 SDs from the average control value were considered abnormal and are in bold. *RMT* resting motor threshold, *AMT* active motor threshold, *MEP* motor evoked potential, *I/O* input–output, *SICI* short-interval intracortical inhibition, *PAS* paired associative stimulation. SICI values indicate the average between SICI at 2 and 4 ms interstimulus intervals. PAS values indicate the mean MEP amplitude recorded at T1, T2, and T3

**Fig. 2:**
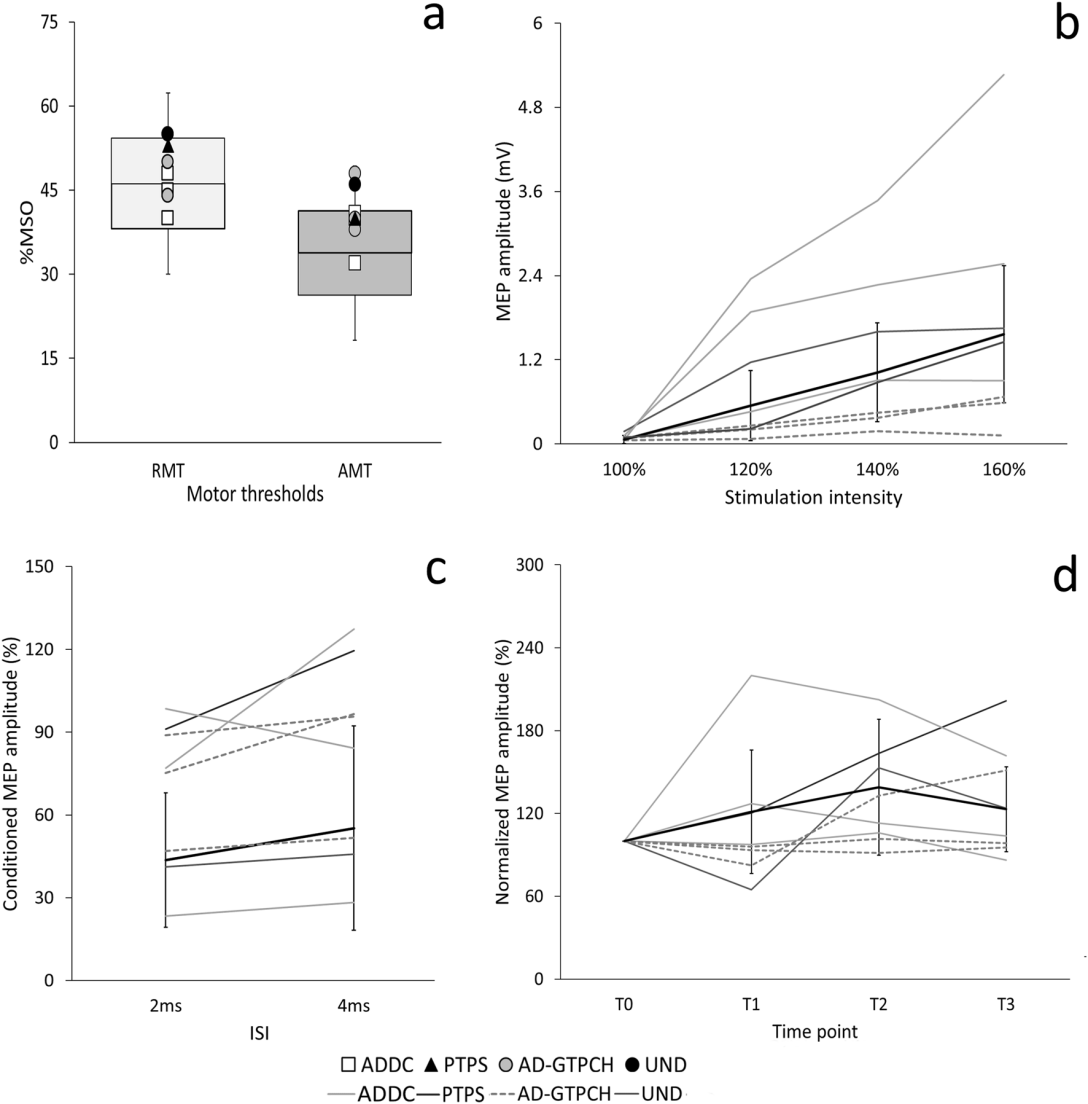
Transcranial magnetic stimulation (TMS) results in patients with monoamine neurotransmitter (NT) disorders and healthy controls (HCs). **a** Resting and active motor thresholds (RMT and AMT), expressed as percentages of the maximal stimulator output (MSO). Horizontal lines denote average values in HCs. Boxes contain the mean value ± 1 standard deviation (SD) of the mean in HCs. Whiskers contain the mean value ± 2 SDs of the mean in HCs. White squares indicate individual data from patients with aromatic amino acid decarboxylase (AADC) deficiency (Patients 1–3). The black triangle indicates data from the patient with 6-pyruvoyl-tetrahydropterin synthase (PTPS) defect (Patient 5). Grey circles indicate individual data from patients with autosomal-dominant GTPCH deficiency (AD-GTPCH) (Patients 6–8). The black circle indicates data from the patient with an early onset parkinsonism associated with a low level of homovanillic acid (HVA) in cerebrospinal fluid but no definitive genetic diagnosis (Patient 9). Patient 4 (tyrosine hydroxylase defect) did not express his consent to undergo TMS procedures. **b** Input–output (I/O) curve of motor-evoked potentials (MEP). The *Y* axis shows the MEP amplitudes (mV); the *X* axis shows the four stimulation intensities (100, 120, 140, and 160% of RMT). HCs are indicated with a thick black line. Patients with AADC deficiency are represented by light grey lines (Patients 1–3). Patient with PTPS defect (Patient 5) is represented by a thin black line. Patients with AD-GTPCH (Patients 6–8) are indicated with dashed grey lines. Patient 9 is indicated by a dark grey line. Error bars denote 2 SDs of the mean in HCs. **c** Short-interval intracortical inhibition (SICI). The *Y* axis shows the ratio between unconditioned and conditioned MEP amplitudes; the *X* axis shows the interstimulus intervals—ISI (2 ms and 4 ms). Error bars denote 2 SDs of the mean in HCs. **d** Course of MEP after the paired associative stimulation (PAS) protocol in the abductor pollicis brevis (APB) muscle. The *Y* axis shows MEP amplitudes normalized to baseline. The *X* axis shows measurements at the four-time points: before PAS (T0) and 5 min (T1), 15 min (T2) and 30 min (T3) after PAS. Error bars denote 2 SDs of the mean in HCs

The most prominent TMS abnormality in patients was higher SICI values, i.e., less intracortical inhibition (5/9, 55.5%). I/O curve slope values were reduced in 3 out of 8 patients (37.5%), meaning that they had a flattened curve as compared to controls. Only one patient (patient 3) showed higher I/O curve slope values, while 50% of patients (4/8) had slope values comparable to controls. Cortical plasticity, as assessed by the response to PAS protocol, was reduced in 3 out of 9 patients (33.3%), increased in two patients (22.2%), and within the normal range in the other 3 patients (Table 3, Fig. 2).

When considering the specific diagnoses, we found that most of the AADC patients (2/3, 66.6%) and the patient with PTPS defect (patient 5) showed reduced intracortical inhibition. I/O curve slope values were normal in these patients. Only one patient with AADC (patient 3) had an increased I/O curve slope and normal SICI. Results regarding cortical plasticity in this subgroup of patients were heterogeneous. Namely, cortical plasticity was reduced in patient 2, enhanced in patients 3 and 5, and normal in patient 1. All AD-GTPCH deficiency patients (100%) had a flatter I/O curve than HCs. In addition, 66.6% of them (2/3) showed higher SICI values, i.e., less inhibition, and a reduced response to PAS protocol.

Finally, patient 9 showed TMS excitability and plasticity measures within the normal range.

### Correlation analysis

We found an inverse correlation between the I/O slope and movement velocity (*R* = − 0.78, *p* = 0.036), and a positive correlation between the I/O slope and CV values (*R* = 0.89, *p *= 0.007), implying that the steeper the I/O curve, the lower the movement velocity and the more irregular the movement rhythm. No other correlations between TMS and kinematic data emerged from the analysis (*R* ranging from − 0.75 to 0.64; *p* ranging from 0.052 to 1, Supplementary Table 1). Finally, no correlation could be found between the biochemical characteristics of the patients at the diagnosis, including pre-treatment concentrations of HVA and 5-Hydroxyindoleacetic acid (5-HIAA) in CSF, and neurophysiological parameters.

## Discussion

The present study was the first to comprehensively investigate the neurophysiological features in patients with monoamine NT disorders, including movement kinematics as well as excitability and plasticity changes of M1. Considering the specific diagnoses, patients with AADC, TH, and PTPS defects all had slow, low amplitude, and irregular movements as compared to controls, without any sequence effect. Conversely, the majority of dopa-responsive dystonia patients showed normal movement performance during finger-tapping. TMS assessment demonstrated that most patients showed reduced M1 intracortical inhibition. Other TMS parameters, including corticospinal excitability and cortical plasticity, were highly variable between patients. Finally, the velocity and rhythm of finger tapping movements correlated with corticospinal excitability changes in all patients, i.e., the higher the corticospinal excitability, the lower the movement velocity and the more irregular the movement rhythm.

The first finding was that patients with AADC, TH, and PTPS defects, who are those with the most severe and generalized defect of dopamine synthesis, showed slow, low amplitude, and irregular movements without any sequence effect during the tapping performance. These findings are similar to those observed in advanced PD patients and in atypical parkinsonisms (APs) but differ from those found in early phases of PD, where the sequence effect is one of the prominent motor abnormality (Postuma et al. 2015; Bologna et al. 2016, 2018). As in advanced PD and APs, the severe reduction in movement amplitude and velocity we here observed hinders the sequence effect during movement repetition, possibly due to a floor effect (Bologna et al. 2016). The lack of sequence effect in monoamine NT disorders supports the emerging idea that bradykinesia features may vary in different conditions characterized by parkinsonism (Bologna et al. 2020). This finding is also in line with the hypothesis that some features of bradykinesia in monoamine NT disorders might not be strictly dependent on dopaminergic loss, but result from a network dysfunction that includes cortical areas (Bologna et al. 2018, 2020; Paparella et al. 2021). As opposed to patients with autosomal recessive defects of dopamine synthesis, those affected by AD-GTPCH had finger tapping kinematics within the normal range. This finding is consistent with the lack of bradykinesia at the time of the study and is in line with previous clinical reports showing that parkinsonian signs in this condition may be mild as compared to other neurological features and that this condition may be favorably influenced by treatment (Segawa 2011). Notably, all patients we studied were investigated after overnight withdrawal (at least 12 h) of their dopaminergic medications. However, since some previous evidence has shown that levodopa effects may still be present several days after the cessation of levodopa in these patients (Nutt and Nygaard 2001), we cannot exclude the possibility that the lack of bradykinesia in this subgroup of patients was due to long-lasting dopaminergic therapy effects. The present result would thus confirm the dramatic and impressive response that this condition has to levodopa.

The second study finding was the evidence of M1 abnormalities as tested by TMS techniques in patients with monoamine NT disorders. We found that the prominent neurophysiological abnormality in patients with AADC, PTPS, and AD-GTPCH defects was the less effective SICI, indicating a reduction in M1 intracortical inhibition. Altered SICI has also been found in PD and APs (Berardelli et al. 2008; Bologna et al. 2018, 2020; Ammann et al. 2020; Guerra et al. 2021) and has been interpreted as a possible compensatory change due to the abnormal influences that the basal ganglia exert on M1 (Cui et al. 2013; Bologna et al. 2020). SICI primarily expresses the activity of γ-aminobutyric acid type A (GABAa) interneurons of M1 (Ziemann et al. 2015). Interestingly, recent studies in rodents have described a direct pallidocortical pathway specifically targeting cortical GABAergic interneurons (Saunders et al. 2015). Thus, similar to what has been observed in animals and humans with PD, it is possible that, due to dopamine depletion, the decreased efferent inhibitory activity of the globus pallidus might lead to M1 disinhibition in patients with monoamine NT disorders (Boraud et al. 1998).

In the present study, we also observed an inverse correlation between movement velocity and rhythm and I/O curve slope in patients with monoamine NT disorders, meaning that the steeper the curve, the slower and more irregular the movement. This result is similar to that observed in patients with PD, who showed a linear relationship between increased M1 corticospinal excitability and movement slowness (Bologna et al. 2018). The present finding supports the hypothesis that M1 dysfunction may contribute to bradykinesia symptoms in conditions characterized by parkinsonism (Berardelli et al. 2001; Bologna et al. 2018, 2020).

Finally, our findings may have relevant implications for the conceptualization of juvenile-onset parkinsonism, for which no consensus on clinical and etiological classification exists (Morales-Briceño et al. 2020; Leuzzi et al. 2021). The finding of similarities in terms of movement amplitude and velocity and intracortical excitability between the disorders with a more profound NT deficit (e.g., AADC, TH, and PTPS deficiency), and advanced PD and APs, may suggest that specific subtypes of primary monoamine disorders have comparable pathophysiological features to the most severe forms of parkinsonism observed in adulthood. This seems not to be the case of AD-GTPCH deficiency, where the normal level of CSF dopamine suggests a milder depletion of this NT at the synaptic level, the neurological impairment is much milder, and the restoration of NT deficit under levodopa supplementation complete. Furthermore, although defective dopamine synthesis is a shared mechanism among different subtypes of primary monoamine disorders and is considered the main pathophysiological issue, other metabolic alterations, such as serotonin depletion in some of them, or disease-specific mechanisms may play a part in the neurophysiological profile and clinical presentation of these disorders. Finally, it should be considered that patients have been assessed in their third decade, while these are the inborn error of metabolism; therefore, some neurodevelopment adaptations or compensatory mechanisms may have influenced the neurophysiological findings.

### Confounding factors and study limitations

Since we did not find any differences in terms of age or gender distribution between patients and HCs, we could also exclude these as potential confounding factors. Concerning the study limitations, the patient sample size is relatively small. However, monoamine NT disorders are rare diseases and most previous neurophysiological studies enrolled a similar number of subjects (Huang et al. 2006; Hanajima et al. 2007). The limited sample size needs to be taken into consideration particularly when interpreting the neurophysiological findings of corticospinal excitability and cortical plasticity, which were highly variable. For example, the flattened I/O curve we found in dopa-responsive dystonia patients and the heterogeneous results regarding M1 plasticity in patients with monoamine NT disorders are difficult to explain and future investigations in larger samples are needed.

## Conclusion

Our study provided the first objective and comprehensive neurophysiological characterization in patients with monoamine NT disorders. Common movement abnormalities were bradykinesia (movement slowness) and hypokinesia (reduced movement amplitude) but not the sequence effect; however, in dopa-responsive dystonia movement parameters were normal or only slightly altered. Again, we demonstrated that in patients with monoamine NT disorders the most prominent TMS finding was reduced intracortical M1 excitability and that the velocity and rhythm of finger tapping movements significantly correlated with corticospinal excitability changes. Further studies are needed to better characterize plasticity changes in patients with monoamine NT disorders. Overall, our data may provide further insight into the pathophysiological mechanisms underlying juvenile parkinsonism due to dopamine deficiency and other parkinsonian conditions.

## Supplementary Information

Below is the link to the electronic supplementary material.

Supplementary file1 (DOCX 15 KB)

## Data Availability

The datasets generated during and/or analysed during the current study are available from the corresponding author on reasonable request.

## Abbreviations

5-HIAA: 5-Hydroxyindoleacetic acid
PTPS: 6-Pyruvoyl-tetrahydropterin synthase
AADC: Aromatic amino acid decarboxylase (AADC)
APB: Abductor pollicis brevis
AMT: Active motor threshold
ANOVA: Analysis of variance
APs: Atypical parkinsonisms
CS: Conditioned stimulus
CV: Coefficient of variation
CSF: Cerebrospinal fluid
EMG: Electromyography
GABAa: γ-aminobutyric acid type A
HCs: Healthy controls
HVA: Homovanillic acid
I/O: Input–output
MDS-UPDRS: Movement disorder society-sponsored revision of the unified Parkinson’s disease rating scale
MEP: Motor-evoked potentials
PAS: Paired associative stimulation
PD: Parkinson’s disease
M1: Primary motor cortex
NT: Neurotransmitter
RMT: Resting motor threshold
SD: Standard deviation
SICI: Short-interval intracortical inhibition
T0: Pre-PAS session
T1: 5 Min post-PAS session
T2: 15 Min post-PAS session
T3: 30 Min post-PAS session
TH: Tyrosine hydroxylase
TMS: Transcranial magnetic stimulation
TS: Test stimulus
