# Supplementary material for: Neurophysiological assessment of juvenile parkinsonism due to primary monoamine neurotransmitter disorders
Source: J Neural Transm (Vienna). 2022 Jul 12;129(8):1011–21. doi: 10.1007/s00702-022-02527-z (PMC9300560; doi:10.1007/s00702-022-02527-z)
Supplement: Supplementary file 1 — Supplementary file1 (DOCX 15 KB) [file 702_2022_2527_MOESM1_ESM.docx]

|  | N. Mov | CV | Amplitude | Velocity | Amplitude slope | Velocity slope | I/O slope | SICI | PAS |
| --- | --- | --- | --- | --- | --- | --- | --- | --- | --- |
| N. Mov | 1.000 |  |  |  |  |  |  |  |  |
|  |  |  |  |  |  |  |  |  |  |
| CV | -0.750 | 1.000 |  |  |  |  |  |  |  |
|  | 0.052 |  |  |  |  |  |  |  |  |
| Amplitude | -0.143 | -0.214 | 1.000 |  |  |  |  |  |  |
|  | 0.760 | 0.645 |  |  |  |  |  |  |  |
| Velocity | 0.393 | -0.714 | 0.643 | 1.000 |  |  |  |  |  |
|  | 0.383 | 0.071 | 0.119 |  |  |  |  |  |  |
| Amplitude slope | 0.857 | -0.821 | 0.107 | 0.536 | 1.000 |  |  |  |  |
|  | 0.014 | 0.023 | 0.819 | 0.215 |  |  |  |  |  |
| Velocity slope | 0.357 | 0.000 | -0.929 | -0.357 | 0.107 | 1.000 |  |  |  |
|  | 0.432 | 1.000 | 0.003 | 0.432 | 0.819 |  |  |  |  |
| I/O slope | -0.393 | 0.893 | -0.357 | -0.786 | -0.571 | 0.179 | 1.000 |  |  |
|  | 0.383 | 0.007 | 0.432 | 0.036 | 0.180 | 0.702 |  |  |  |
| SICI | 0.036 | -0.286 | 0.143 | 0.500 | 0.107 | 0.143 | -0.393 | 1.000 |  |
|  | 0.939 | 0.535 | 0.760 | 0.253 | 0.819 | 0.760 | 0.383 |  |  |
| PAS | 0.000 | 0.429 | -0.464 | -0.429 | 0.071 | 0.393 | 0.536 | -0.429 | 1.000 |
|  | 1.000 | 0.337 | 0.294 | 0.337 | 0.879 | 0.383 | 0.215 | 0.337 |  |

**Supplementary table 1. Correlation analysis.** Shown are the R and p values from the correlation analysis between kinematic and transcranial magnetic stimulation (TMS) data.
